# Investigation of the optimum location of external markers for patient setup accuracy enhancement at external beam radiotherapy

**DOI:** 10.1120/jacmp.v17i6.6265

**Published:** 2016-11-08

**Authors:** Payam Samadi Miandoab, Ahmad Esmaili Torshabi, Saber Nankali

**Affiliations:** ^1^ Department of Electrical and Computer Engineering Kerman Graduate University of Advanced Technology Kerman Iran

**Keywords:** optimum location, external markers, input selection algorithm, patient setup, external beam radiotherapy

## Abstract

In external beam radiotherapy, one of the most common and reliable methods for patient geometrical setup and/or predicting the tumor location is use of external markers. In this study, the main challenging issue is increasing the accuracy of patient setup by investigating external markers location. Since the location of each external marker may yield different patient setup accuracy, it is important to assess different locations of external markers using appropriate selective algorithms. To do this, two commercially available algorithms entitled a) canonical correlation analysis (CCA) and b) principal component analysis (PCA) were proposed as input selection algorithms. They work on the basis of maximum correlation coefficient and minimum variance between given datasets. The proposed input selection algorithms work in combination with an adaptive neuro‐fuzzy inference system (ANFIS) as a correlation model to give patient positioning information as output. Our proposed algorithms provide input file of ANFIS correlation model accurately. The required dataset for this study was prepared by means of a NURBS‐based 4D XCAT anthropomorphic phantom that can model the shape and structure of complex organs in human body along with motion information of dynamic organs. Moreover, a database of four real patients undergoing radiation therapy for lung cancers was utilized in this study for validation of proposed strategy. Final analyzed results demonstrate that input selection algorithms can reasonably select specific external markers from those areas of the thorax region where root mean square error (RMSE) of ANFIS model has minimum values at that given area. It is also found that the selected marker locations lie closely in those areas where surface point motion has a large amplitude and a high correlation.

PACS number(s): 87.55.km, 87.55.N

## I. INTRODUCTION

In external beam radiotherapy, the final goal is to deliver 3D uniform dose to tumor volume while minimizing the dose to healthy surrounding tissues at the same time. In recent years, many efforts have been done on two substantial challenging issues to achieve a successful radiotherapy: 1) tumor localization and delineation at treatment planning system and 2) dose delivery at beam irradiation system.^(1)^ But degree of a success treatment is strongly depending on the accuracy of tumor localization as a major part of treatment planning process. Moreover, this issue will be a serious concern for tumors located at thorax region due to semiregular motions caused by heartbeat, gastrointestinal, and especially breathing phenomena known as intrafractional organ motion error. Intrafractional organ motion error may lead to a significant uncertainty of tumor localization, which reduces treatment quality by increasing a great amount of dose received by surrounding tissues, which causes side effects at thoracic regions.^(1)^ To compensate the effect of intrafraction motion error and to minimize target localization uncertainty, several studies have been performed, while some of them are now clinically available.^2^, ^3^, ^4^, ^5^, ^6^ An older strategy associated with intrafractional motion error is defining a larger margin around gross target volume that includes tumor volume and its motion trajectory as internal target volume (ITV).^(7)^ By this approach, a great amount of prescribed dose will be received by normal tissues at near the ITV region that may produce serious side effects. Other clinically available strategies to compensate tumor motion errors are: 1) breath‐holding,^8^, ^9^, ^10^, ^11^ 2) real‐time tumor tracking,^(12)^ and 3) respiratory gating.^(13)^ Before treatment in external beam radiotherapy, target volume alignment in front of the therapeutic beam in the pretreatment step, known as patient geometrical setup, must be considered seriously.^14^, ^15^, ^16^, ^17^, ^18^, ^19^, ^20^, ^21^, ^22^, ^23^, ^24^ In order to manage respiratory motion, several surrogates systems such as: spirometer,^5^, ^25^ strain gauge,^(25)^ time‐of‐flight cameras,^(26)^ and external markers^(27)^ are utilized as a dataset providers for patient setup. It should be noted that these devices with synchronously captured internal dataset, based on correlation model, may also use during treatment for real‐time tumor motion tracking.^12^, ^13^ In addition, the success of patient setup and then tumor motion tracking is strongly affected by the number and location of external markers. In most clinical applications while treating real patients, the location of external markers is chosen empirically; that is operator‐dependent and can be a constraint due to missing optimum location. Moreover, few studies have been performed to mathematically investigate optimum location of external markers at the pretreatment step while patient positioning must be performed accurately. Dong et al.^(15)^ performed a mathematical study to investigate optimum markers’ location during treatment using Bregman distance‐based algorithm.

In the present study to find the best location of external markers for patient setup, two nonlinear strategies based on a) canonical correlation analysis (CCA),^(28)^ and b) principal component analysis (PCA)^(29)^ are proposed as an input selection algorithms. These algorithms were chosen due to their proven intrinsic robustness at data mining. The input selection concept was introduced by Samadi‐Miyandoab et al.^(30)^ as a dimensionality reduction strategy at data mining procedure. In this method, irrelevant features are detected and then removed to yield most effective reduced dataset for predictive model construction. Moreover, a comparative study is done between two proposed algorithms taking into account the advantages and weakness points of each method that was comprehensively assessed in our recent study.^(30)^ To implement of the proposed method, an adaptive neuro‐fuzzy inference system (ANFIS) is utilized as responsible for aligning tumor volume in front of the therapeutic beam by giving tumor motion information. In fact, the proposed CCA and PCA input selection algorithms act as provider of ANFIS input and the best patient geometrical setup is estimated by means of an ANFIS model. Therefore, ANFIS is fed by motion information of those external markers that were already selected by proposed CCA and PCA input selection algorithms. In this study, the ANFIS model is proposed due to its robustness to combine the abilities of fuzzy systems with the numeric power of neural adaptive network systems. Moreover, since the degree of variability of our dataset is very high in different patients, ANFIS is optimal to estimate the best location of external markers with less error.

The required dataset used in this work has been extracted using the 4D XCAT anthropomorphic phantom developed by Dr. W.P. Segars and provided by NURBS. This validated phantom simulates the 3D anatomical shape of different organs of human body with acceptable complexity and models motion of dynamic organs located in thorax region to mimic real respiratory and heart beat motion patterns.^31^, ^32^ In order to assess optimum location of external markers, nine subregions have been defined uniformly on the thorax region of the patient body. Comprehensive studies were done during our recent studies, in which different aspects of six‐degrees‐of‐freedom rototranslation of this phantom was investigated.^30^, ^33^ Furthermore, we utilized the database of four real patients with lung cancers undergoing radiation therapy to validate the simulated procedure.

In order to test the performance of proposed input selection algorithms at finding optimum markers location, root mean square error (RMSE) of ANFIS output was considered as a metric for quantitative evaluation. In this way, the RMSE of ANFIS model output for each segment that represent marker placement indicates the “importance degree” of that segment as optimum location. Moreover, the performance of the proposed strategy is compared with empirical methods that use clinically.^(30)^ Final analyzed results represent that the implementing of these input selection algorithms can significantly improve patient geometrical setup errors in regard with to the conventional method performed empirically by operator. It is also found that the selected marker locations have a large amplitude and frequency, and a high correlation.

## II. MATERIALS AND METHODS

### A. Database generation and its properties

In order to provide a validated dataset, a simulation study was performed using the NURBS‐based 4D XCAT anthropomorphic phantom that can model the shape and structure of complex organs in human body along with motion of dynamic organs such as are involved in breathing and heartbeat.^(34)^ This phantom was chosen due to its validation and commercial availability.^(31)^ XCAT is quite robust to simulate the human body with multiple resolutions and various anatomies due to combining the advantages of pixel‐based and geometry‐based analyses. This phantom enables user to change functional variables that control respiration, in order to generate deformable 4D CT models according to the real conditions of a typical patient that must be simulated. The main controllable parameters are: 1) motions of beating heart only, respiration only, or combined mode; 2) maximum diaphragm motion; and 3) maximum anterior–posterior expansion of the chest wall.^(34)^ In this study, five different respiratory cycles were generated with reasonable breathing amplitude and frequency to mimic real respiratory patterns (Table 1). For instance, maximum anterior–posterior expansion of chest wall and time of respiratory period were determined using respiratory motion signals of real patients treated with the CyberKnife Synchrony system (Accuray Inc., Sunnyvale, CA) at Georgetown University Medical Center (Washington, DC).

According to extracted database from the XCAT phantom, due to negligible displacement in left‐right (LR) direction, this dimension was eliminated from total database and remaining motion data included both anterior–posterior (AP) and superior–inferior (SI) directions. Time interval between data acquisition steps required for each frame was assumed to be 25 ms. The proposed strategies were validated through simulated procedures by using the 4D CT database acquired from four patients as reference (Table 2).

**Table 1:** Characteristics of five different respiratory cycles created by XCAT phantom

*Maximum Anterior–Posterior Expansion of Chest Wall (cm)*	*Maximum Diaphragm Motion (cm)*	*Time of Respiratory Period (sec)*	*Breathing Cycle Number*
1.2	2	5	1
0.7	1.7	5	2
0.5	1.2	4	3
1.3	2.2	6	4
1	1.8	5.5	5

**Table 2:** Patients’ 4D CT data

*Patient*	*Image Dimension*	*Pixel Dimension (mm)*
Patient #1	512×512×169	0.97×0.97×2
Patient #2	512×512×170	0.87×0.87×2
Patient #3	512×512×187	0.78×0.78×2
Patient #4	512×512×161	1.17×1.17×2

**Figure 1:**
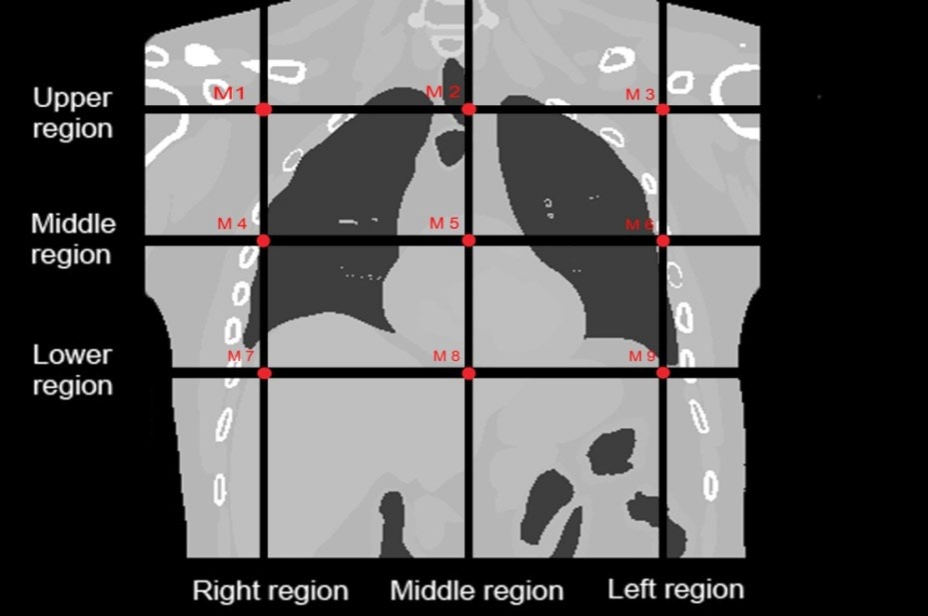
The location of each external marker on the surface of phantom body. M1 = right upper lobe, M2 = middle upper lobe, M3 = left upper lobe, M4 = right middle lobe, M5= xiphoid, M6= left middle lobe, M7 = right lower lobe, M8 = navel upper, M9 = left lower lobe.

In order to assess optimum location of external markers, nine subregions have been defined uniformly on the thorax region of patient body (Fig. 1). In other words, nine typical points from nine sub‐regions were chosen onto the surface of the chest and abdominal regions, each of them representing external surrogates. The scheme of depicted points started from abdominal region with averagely 5 cm distance in vertical and horizontal direction (Fig. 1). As seen in this figure, the proposed spatial scheme of given points was divided into three areas: upper region, middle region, and lower region, respectively. It should be noted that the performance of the proposed strategy is compared with empirical methods, which are clinically available and suggested by prior studies.^30^, ^33^

### B. The methodology of CCA, PCA selective algorithms, and the ANFIS model

The CCA and PCA input selection methods are introduced as useful available tools required at the data preprocessing stage of each data mining, in which the number of data points belonging to the input dataset is reduced by removing irrelevant, redundant, or noisy data points. Therefore, most effective and remarkable data points obtained in this strategy can improve mining performance such as predictive accuracy and final results may obtained in a better assessment condition. In order to find the best location of external markers, two properties of the data set, 1) the maximum correlation coefficient and 2) minimum variance of external markers’ location, are considered among total available markers that are selected by CCA and PCA algorithms. To do this, the MATLAB software package (The MathWorks Inc., Natick, MA) was utilized. When optimum locations were chosen from among total markers locations, the motion information of selected markers are used as an input file of the ANFIS model for verification of patient geometrical setup. On the other hand, the output of the ANFIS model can be used to discover two important factors: 1) the performance of each input selection algorithm, and 2) the importance degree of each marker location.

Canonical correlation analysis (CCA) is a means to measure a linear relationship between two multidimensional variables. In statistics, CCA is a way of making sense of cross covariance matrices. If we have two vectors $X\;=\;\left(X_{1},\;\ldots ,\;X_{n}\right)$ and $Y\;=\;\left(Y_{1},\;\ldots ,\;Y_{m}\right)$ of random variables, and there are correlations among the variables, then canonical correlation analysis will find linear combinations of the $X_{i}$ and $Y_{j}$ which have maximum correlation with each other. The CCA is optimal way to respect correlations and at the same time to find a corresponding correlation, in which the correlation matrix between the variables is diagonal and the correlations on the diagonal are maximized. In fact, the dimensionality of these new bases is equal to or less than the smallest dimensionality of the two variables.^(35)^ By applying CCA algorithm, a small amount of data will be lost when more than 90% of canonical correlation of all markers is covered. Principal component analysis (PCA) is a statistical procedure that uses an orthogonal transformation to convert a set of observations of possibly correlated variables into a set of values of linearly uncorrelated variables called principal components. PCA can be thought of as fitting N‐dimensional ellipsoid to the data, where each axis of the ellipsoid represents a principal component. The number of principal components is less than or equal to the number of original variables. This transformation is defined in such a way that the first principal component has the largest possible variance; that is, accounts for as much of the variability in the data as possible, and each succeeding component in turn has the highest variance possible under the constraint that it is orthogonal to the preceding components. The resulting vectors are an uncorrelated orthogonal basis set. The principal components are orthogonal because they are the eigenvectors of the covariance matrix, which is symmetric. PCA is sensitive to the relative scaling of the original variables. In other hand, PCA was implemented for three X, Y, and Z variables in order to reduce the number of inputs, and then the first principal component was utilized. After transformation, this transformation is defined in such a way that the first principal component has the largest possible variance, and each succeeding component in turn has the highest variance possible under the constraint that it is orthogonal to the preceding components.^(36)^ Moreover, in order to reduce the numbers of inputs to select markers, PCA was implemented and then the first principal component was utilized. By applying PCA algorithm, a small amount of data will be lost when the first component of all markers covers more than 90% of variance. PCA transforms the 3D motion data of external markers into a mono‐dimensional signal, by projecting the three‐dimensional coordinates in the principal component space.^(29)^

We developed the ANFIS model by implementing the fuzzy logic toolbox of the MATLAB.^(37)^ ANFIS is presented as a powerful tool in modeling numerous processes by combining the abilities of a fuzzy system with the numeric power of a neural network system. The fuzzy inference system of the ANFIS is based on Sugeno‐type and membership functions generated by FCM data clustering algorithm in Gaussian form. In this study, we used the robustness of the ANFIS particularly as a correlation model for verification of patient geometrical setup where similar calculations with normal mathematical methods would be difficult or with less accurate. More information about the ANFIS model is shown in Table 3. Our ANFIS correlation model must be trained using dataset at synchronized form in training step. After configuration, the model is able to realign patient position in front of the therapeutic beam.

**Figure 2:**
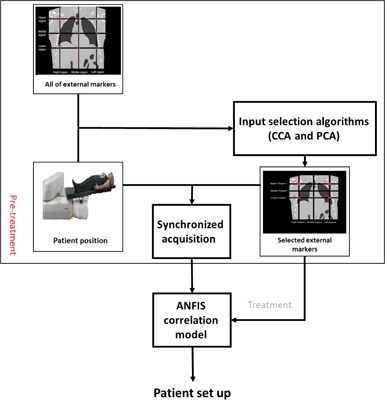
workflow of required process on input selection algorithms by CCA and PCA with ANFIS correlation model to verification of geometrical setup.

Figure 2 shows a schematic layout of 1) the PCA and CCA in data processing as an input selection algorithm, and 2) ANFIS correlation model configuration at pretreatment step and model performance during treatment. As depicted, when optimum external makers were chosen automatically, their motion data was synchronized with patient position for verification of patient setup at the pretreatment step. In fact, the proposed CCA and PCA input selection algorithms provide the ANFIS input dataset and the best patient geometrical setup is estimated from ANFIS model output.

**Table 3:** The structure of the adaptive neuro‐fuzzy interference system (ANFIS) model

*Parameter*	*Type*
And Method	Product of Elements
OR Method	Probabilistic OR
Implication Method	Product of Elements
Aggregation Method	Sum of Elements
Defuzzification Method	Weighted Average
Input Membership Function	Gaussian

## III. RESULTS

In order to test and evaluating the performance of proposed strategies for improving patient setup, the results were expressed by computing the root mean square error (RMSE) between benchmark data points and ANFIS model output as follows:

$$RMSE=\sqrt{\frac{1}{N}\sum_{i=1}^{N}{\left(A_{i}-P_{i}\right)}^{2}}$$

where *N* is the number of predicted samples, $A_{i}$ is $i_{\text{th}}$ data point representing real position information and $P_{i}$ is the $i_{\text{th}}$ predicted value of position given by ANFIS.

**Figure 3:**
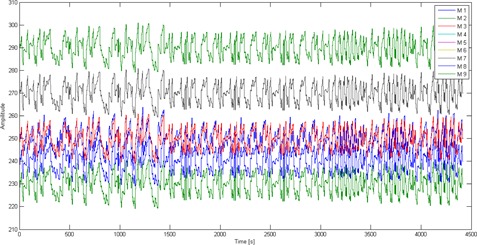
The maximum and minimum signal in each region of Patient 2 of the skin surface during the respiratory cycle.

Generally, each subregion of thorax and abdomen (Fig. 2) has its own degree of importance due to two major factors: 1) correlation of belonged external markers with corresponding reference configuration, and 2) its motion amplitude during breathing amplitude. This importance degree is increased when a large number of external markers from a typical subregion are selected by our proposed CCA and PCA input selection algorithms. Table 4 demonstrates the average amplitude and frequency between the external markers and reference data points over all four given patients. The amplitude varies from 2,200 to 3,100 for all sessions. In addition, the maximum and minimum signal at each subregion of patient number 2 is observed in Fig. 3.

Based on the information emerging from Fig. 3, the importance degree belongs to the right lower lobe (M7) and left lower lobe (M9), where the maximum signals are extracted. At the same time, the minimum signal belongs to XIPHOID (M5) and navel upper (M8) that represent the negligible degree of importance. According to the results shown in Fig. 3 and Table 4, the i) maximum, ii) minimum, iii) average, and iv) frequency parameters obtained from each subregion (provided by each external marker) are different for each patient, uniquely. Moreover, these four parameters are highly affected by marker location. The maximum and minimum signal belongs to M7 and M5 markers, respectively.

As seen from Table 4 and Fig. 3, the number of optimum external markers for a given region is selected by input selection algorithms. The selection process is repeated using CCA and PCA methods and then the summation of all chosen external markers at each subregion is calculated (Table 5). This summation value represents the importance degree of each region. At the CCA and PCA algorithms, which the high accuracy of high correlation coefficient and minimum standard deviations were taken into account. As seen in Table 5, M1, M3, M7, and M9 were selected. Furthermore, among four real patients, the averages of correlation coefficients and standard deviations are as: 0.98 ± 0.18 (M1), 0.67 ± 0.23 (M2), 0.97 ± 0.20 (M3), 0.97 ± 0.32 (M4), 0.67 ± 0.21 (M5), 0.96 ± 0.30 (M6), 0.98 ± 0.21 (M7), 0.67 ± 0.21 (M8), and 0.97 ± 0.19 (M9). Correlation coefficients calculated for M4 and M6 are closer to M1, M3, M7, and M9, but with larger fluctuation. Moreover, correlation coefficients of M2, M5, and M8 are relatively small, but in an acceptable range. Therefore, it's worth mentioning that the amount of correlation coefficient is affected by breathing patterns and external markers location. The other results can be observed in pretreatment step from Table 4; that is, maximum amplitude and frequency in the right middle lobe (M4), left middle lobe (M6), right lower lobe (M7), and left lower lobe (M9) respectively, but, based on Table 5, represented by using CCA and PCA methods, the selected markers are M1, M3, M7, and M9 — high accuracy of high correlation coefficient and minimum standard deviations. In other words, the average of the correlation coefficients calculated and standard deviations for four real patients indicated the markers selected (M1, M3, M7, and M9) monitors accuracy of input selection algorithms.

**Table 4:** The average amplitude and frequency between the markers and reference point in the four patients

	*M1*		*M2*		*M3*		*M4*		*M5*		*M6*		*M7*		*M8*		*M9*	
	*Am*	*F*	*Am*	*F*	*Am*	*F*	*Am*	*F*	*Am*	*F*	*Am*	*F*	*Am*	*F*	*Am*	*F*	*Am*	*F*
Patient #1	2500	74	2300	73	2550	74	3000	75	2500	74	3000	75	2800	74.5	2450	73.5	2800	74.5
Patient #2	2500	74	2300	73	2550	74	3050	75	2550	74	3050	75	2700	75	2500	73.5	2900	75
Patient #3	2200	73	2200	73	2200	73	2700	75.5	2550	75.5	3100	77	2400	74	2200	73	2300	73
Patient #4	2300	73.5	2200	73	2200	73	2800	75	2400	74	2800	75	2600	74	2300	73	2500	74

M1 = marker one, M2 = marker two, M3 = marker three, M4 = marker four, M5 = marker five, M6 = marker six, M7 = marker seven, M8 = marker eight, M9 = marker nine, Am = Amplitude, F = Frequency

Moreover, we compared our proposed strategy with currently used empirical methods and investigated interfraction motion error.

**Figure 4:**
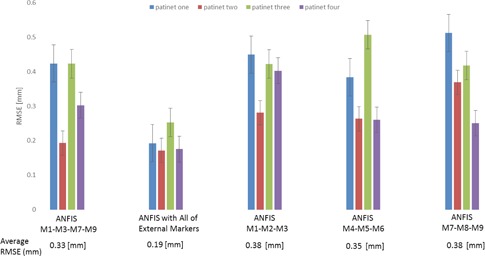
The results illustrates RMSE calculated between implementing input selection models in combination with ANFIS model, ANFIS model using all‐markers dataset and ANFIS model, by using motion information of given external markers at each upper, lower, and middle region and corresponding reference configuration.

The result of CCA and PCA input selection algorithms used to select optimum external markers (Fig. 4). The motion dataset of selected markers was utilized as an input file for the ANFIS model automatically, for real‐time verification of the geometrical setup. Figure 4 illustrates RMSE calculated between ANFIS model output and corresponding reference data points.^(30)^ As shown, a comparison was done in different modes: 1) implementing input selection models in combination with ANFIS model, 2) ANFIS model by using all markers dataset, and 3) ANFIS model by using motion information of given external markers at each upper, lower, and middle regions, respectively.

**Table 5:** Result of input selection algorithms (CCA and PCA model) and selected external markers

	*Average Correlation Coefficient*		
*Number of Markers*	*CCA Model*	*PCA Model*	*Selected Markers*
M 1	0.99	0.99	a
M 2	0.57	0.67	b
M 3	0.99	0.99	a
M 4	0.72	0.96	b
M 5	0.46	0.67	b
M 6	0.72	0.96	b
M 7	0.99	0.99	a
M 8	0.57	0.67	b
M 9	0.99	0.99	a

a Selected external markers.

b Unselected external markers.

## IV. DISCUSSION

In radiation treatments, tumors located in thorax region of a patient body move mainly due to respiration. This motion is problematic during therapeutic beam irradiation, and may result in undesirable dose distribution in tumor volume and also deliver high doses to healthy non‐target tissues. The motion error is due to 1) breathing phenomena (known as intrafractional error), and 2) patient positioning (known as interfractional error) while aligning tumor volume against the therapeutic beam. To compensate intrafractional motion error, several strategies may be implemented, such as 1) breath‐holding, 2) real‐time target tracking, and 3) respiratory gating.^(30)^ At the pretreatment step, serious concerns arise due to interfractional motion error between each fraction of the treatment process that reduce the accuracy of patient setup. In modern radiotherapy, the success of a treatment strongly depends on the accuracy of patient geometrical setup. One strategy for patient positioning is using external markers on the thorax region of the patient body. Therefore, the markers’ location in external radiotherapy is important and challenging during patient setup. In most clinical applications, the location of external markers is chosen empirically; that is, operator‐dependent.

In this study to find the optimum spatial pattern of external markers onto thorax skin, two common available nonlinear strategies were proposed to select the best location of external markers, intelligently. These strategies are on the basis of CCA and PCA input selection algorithms that work by means of maximum correlation coefficient and minimum variance parameters over total number of available locations for external markers. The proposed methods were tested through simulation studies using the verified NURBS‐based 4D XCAT anthropomorphic virtual phantom to provide the required dataset as if from the breathing pattern of a real patient. Moreover, the proposed strategy was validated using the reference 4D CT dataset from four real patients. Nine external markers on the thorax surface were taken into account by this phantom and real patient to simulate external motions along with a corresponding reference configuration (Fig. 1). The motion dataset of selected markers were utilized for feeding an ANFIS model to estimate patient geometrical setup, accordingly.

Based on the results emerged from Fig. 3 and Table 4, the most important subregions as optimum marker placement are right upper lobe, left upper lobe, right lower lobe, and left lower lobe, chosen by the proposed input selection algorithms. In other words, these chosen subregions have the highest importance degree, whereas middle areas with negligible values are far away from participation as external markers location. Also, based on the results reported by Ehrhardt et al.^(38)^ and Werner et al.,^(39)^ the maximum displacement of the skin surface during the respiratory cycle of patient occurs in the border areas.

Correlation between the motion of external markers and reference point may be affected by several factors, including patient characteristics, marker locations, and breathing pattern. It should be noted that a single external marker cannot provide sufficient and reliable information for patient setup, while a composite signal generated from the motion information of multiple external makers may provide an excellent and reliable setup with less error as demonstrated in this work. Furthermore, the importance degree of each subregion is increased when a large number of external markers from a typical area are selected by our proposed input selection algorithms. As shown in Table 4 and Fig. 3, the selected M4, M6, M7, and M9 markers move with high amplitudes and frequency. But based on results of the CCA and PCA algorithms reported at Table 5, the external markers M1, M3, M7, and M9 are in the most important sub‐regions with high correlation and minimum variance. Moreover, it's worth mentioning that the border areas have the highest degree of importance, whereas middle areas with lower values are far away from participation as external markers. The performance accuracy of the ANFIS model configured by different external markers chosen by different input selection algorithms represents the best opportunity for finding optimum external markers. Therefore, it is concluded that the input selection algorithms work reasonably well for finding the best location of external markers, and may be preferred to the conventional clinical method that is done empirically, leading to minimization of interfraction motion errors in real patients.

## V. CONCLUSION

In this study, we used a 4D XCAT phantom and real patients to investigate input selection algorithms to find optimum external marker locations that have the best correlation with corresponding reference configuration. In addition, the results of the model were compared with our previous reports concerning patient positioning at external beam radiotherapy.^(30)^ Based on Table 5, we found that the input selection algorithms selected best locations of external markers (M1, M3, M7, and M9) during patient setup using ANFIS algorithm. Also, the technique was validated through simulated activities by using reference 4D CT data acquired from four subjects.

## ACKNOWLEDGMENTS

The authors acknowledge Dr. William Paul Segars, Dr. David Surratt, and Sonja Dieterich for providing access to the anthropomorphic XCAT phantom, 4D CT data of four real patients, and clinical CyberKnife database, respectively.

## COPYRIGHT

This work is licensed under a Creative Commons Attribution 3.0 Unported License.
